# Sex-dependent plasticity of adult neural tissue in response to damage

**DOI:** 10.1242/dev.204945

**Published:** 2026-02-20

**Authors:** Marina Recatalà-Martinez, Manel Bosch, Pedro Gaspar, Alessandro Mineo, Santiago Rios, Irene Miguel-Aliaga, Marta Morey

**Affiliations:** ^1^ Departament de Genètica, Microbiologia i Estadística, Facultat de Biologia, Universitat de Barcelona, Barcelona 08028, Spain; ^2^ Unitat de Microscòpia Òptica Avançada, Centres Científics i Tecnològics de la Universitat de Barcelona (CCiTUB), Barcelona 08028, Spain; ^3^ The Francis Crick Institute, London NW1 1AT, UK; ^4^ Human Genetics and Disease, Institut de Biomedicina de la Universitat de Barcelona (IBUB), Barcelona 08028, Spain

**Keywords:** Neural tissue, Adult plasticity, Sex differences

## Abstract

The plasticity of intact adult neural tissue in the vicinity of neural damage helps restore circuit function. Much remains to be learned about the mechanisms regulating this process and the reported sex differences in recovery outcomes. Here, we present the fly gut and its innervation as a simplified model to address these questions. We show that ingestion of damaging agents triggers a reversible increase in adult enteric neural tissue in females, consistent with growth rather than neurogenesis. This growth can be influenced by gut-derived reactive oxygen species, as suggested by suppression with an antioxidant. Interestingly, males do not display neural plasticity, and masculinization of neurons in females suppresses damage-dependent neural growth. Conversely, feminizing male neurons does not confer plasticity, suggesting that sex-specific cues from surrounding tissues may be required for this response. Blocking plasticity reduces the dextran sulfate sodium-induced increase in defecation and further shortens survival, indicating that female-specific neural plasticity supports both gut function and viability. Together, these findings establish a physiological model to dissect cellular, molecular and sex-dependent regulators of adult neural plasticity relevant to circuit repair.

## Teaser

**Summary:** The *Drosophila* gut can be used as a simple system to explore how adult neural tissues differ between sexes in their capacity for plasticity.

## INTRODUCTION

Neural plasticity involves two key mechanisms for recovery after injury: axonal regeneration and structural plasticity of intact neural tissue. While axonal regeneration has been widely studied (Mahar and Cavalli, 2018; Tedeschi and Bradke, 2017), modulating the plasticity of remaining healthy neural tissue offers an alternative route to restore function (Gao et al., 2022; Petersen et al., 2022). Clinical evidence shows that this form of plasticity supports functional recovery and circuit reorganization after acute damage such as stroke (Cirillo et al., 2020; Murphy and Corbett, 2009; Nudo, 2003; Sampaio-Baptista et al., 2018) or multiple sclerosis episodes (Ksiazek-Winiarek et al., 2015; Prosperini et al., 2015). Notably, recovery outcomes show sex-specific differences (Turtzo and McCullough, 2008; Yu et al., 2015), suggesting variability in plasticity capacity (Hyer et al., 2018; Kirby et al., 2024).

Despite these observations, the molecular mechanisms regulating plasticity in intact adult neural tissue remain poorly understood, partly due to the challenge of studying healthy and damaged neurons in close proximity. To address this, we developed a model that examines how intact neurons respond to non-neural tissue damage, using peripheral innervation as an accessible system. This enables controlled injury and analysis of plasticity in a physiologically relevant yet simplified context.

We used the digestive tract of *Drosophila melanogaster* as a model. As in mammals, the fly gut is structurally and functionally compartmentalized (Buchon et al., 2013; Lemaitre and Miguel-Aliaga, 2013; Marianes and Spradling, 2013). Its epithelium includes intestinal stem cells (ISCs), enteroblasts (EBs), enterocytes (ECs) and enteroendocrine (EE) cells. The gut is wrapped in muscle, oxygenated by tracheae, and innervated by neurons from the central nervous system, corpora cardiaca and hypocerebral ganglion (HCG) (Kuraishi et al., 2015; Miguel-Aliaga et al., 2018). Most neurites target visceral muscle, with some extending to the epithelium (Cognigni et al., 2011; Cui et al., 2024 preprint; Kenmoku et al., 2016; Petsakou et al., 2023). Unlike mammals, innervation is regionally restricted, enabling targeted analysis. Recent studies show that gastrointestinal neurons respond to microbiota, nutrients, aging and reproductive state in flies and mammals (Ameku et al., 2020; Hadjieconomou et al., 2020). While functional plasticity in the posterior fly midgut during recovery has been documented (Petsakou et al., 2023), mechanisms of structural plasticity after gut damage – and their modulation by biological sex – remain unknown. The system presented here provides a unique opportunity to investigate how sex and tissue context shape neural plasticity, and to uncover molecular pathways linking damage signals to structural remodeling.

## RESULTS AND DISCUSSION

### Intestinal damage induces reversible neural plasticity

**Fig. 1.**
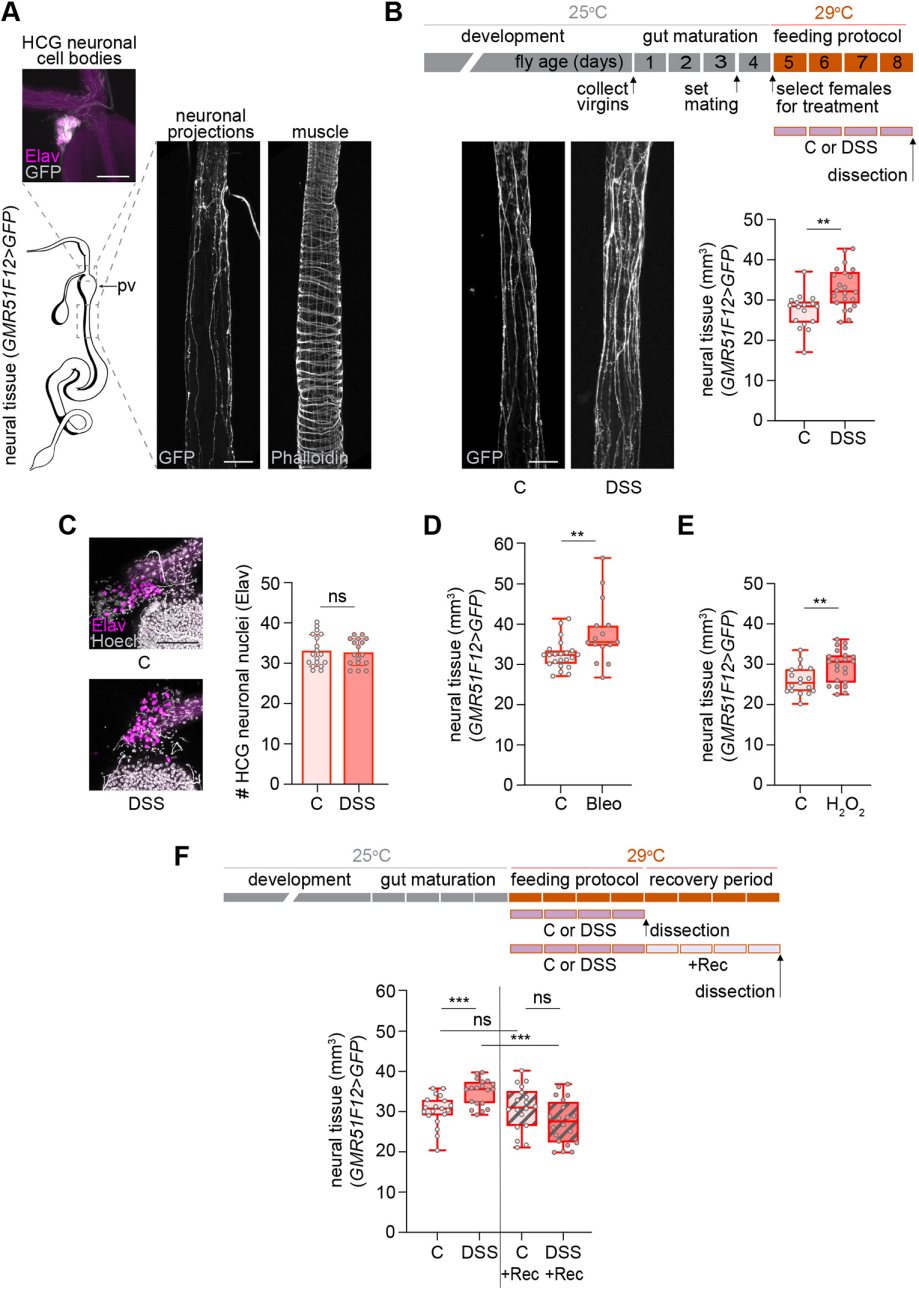
**Neural plasticity induced by gut damage resolves after recovery.** (A) Adult gut drawing highlighting two regions with corresponding confocal *z*-projections: above the proventriculus (pv), showing neuronal nuclei (magenta, Elav) and membranes (gray, GFP) of the HCG; and the anterior midgut, showing neuronal processes (GFP) and muscle fibers (phalloidin). (B) Detailed experimental timeline schematic with top rectangles representing 1 day each and reflecting the age of the flies (gray and orange) and bottom purple rectangles represent the duration of the feeding protocol or any other additional period after that (recovery; light purple in F). Confocal *z*-projections of anterior midgut innervation in control or DSS-fed females (C or DSS) with neural tissue quantification. A red outline in the box and whisker plots or in the bar graphs indicates female. (C) HCG *z*-projections of control and DSS-fed females stained with Hoechst (gray, which also accumulates in some tracheal branches) and for Elav (magenta) to quantify the number of neuronal nuclei. (D,E) Neural tissue quantification in control versus bleomycin-fed (D) and control versus H_2_O_2_-fed (E) females. (F) Feeding and recovery schematic (top); neural tissue quantification after protocol and recovery (Rec) (bottom). Scale bars: 50 μm. **P<0.01, ***P<0.001; ns, not significant (ANCOVA for B,D-F; unpaired *t*-test for C).

To quantify anterior midgut innervation, we used a driver and reporter combination labeling neuronal processes with native GFP. This prevented background signal from secondary antibodies and provided a high signal-to-noise ratio, facilitating faithful neural process quantification as GFP signal. Because general neuronal markers, such as Elav (Chen et al., 2016; Holsopple et al., 2022; Titos et al., 2023) or n-Synaptobrevin (Weaver et al., 2020) also label EEs, we used the *GMR51F12-GAL4* (Jenett et al., 2012; Min et al., 2021; Olds and Xu, 2014) and the specific *nSyb.S-GAL4* (Weaver et al., 2020) drivers, which do not. Morphological landmarks in whole-mount preparations, combined with phalloidin co-staining to visualize the surrounding musculature, enabled consistent imaging of the same digestive tract region and its associated neural innervation (Fig. 1A). Using 3D reconstruction and image analysis, we obtained volume quantifications for both the digestive tube and neural tissue. Scaling growth is a prevalent phenomenon in the peripheral nervous system, where sensory and motor neurons must adjust the size of their arborizations according to the area of their target tissues to maintain their functionality (Bentley and Toroian-Raymond, 1981; Bucher and Pflüger, 2000; Lee and Stevens, 2007; Menon et al., 2013). Measuring the volume of the digestive tube enabled us to use statistical analysis methods that accounted for and removed any potential influence of digestive tube size variation in adults (driven by treatment or genetic background) on neural tissue quantification comparisons and to distinguish scaling growth from neural plasticity tissue growth.

To damage the gut, we fed flies with dextran sulfate sodium (DSS). DSS is a polymer that induces colitis in mammals (Yang and Merlin, 2024) and has been used in *Drosophila* to study the cellular mechanisms and molecular pathways that regulate ISC proliferation during regeneration (Jiang et al., 2016; Tian et al., 2022). When flies ingest DSS, it crosses the epithelium, expanding the basement membrane sheet and altering muscle morphology (Howard et al., 2019). The resulting change in biomechanical forces in the niche is thought to activate ISC division (Howard et al., 2019). DSS feeding also results in trachea sprouting (Perochon et al., 2021), which is necessary for ISC proliferation (Medina et al., 2025; Perochon et al., 2021; Tamamouna et al., 2021). Interestingly, basement membrane and muscle defects, as well as trachea remodeling, are reversible after a recovery period (Howard et al., 2019; Perochon et al., 2021; Tamamouna et al., 2021). We wondered whether adult neurons were plastic and could also respond to gut damage.

**Fig. 2.**
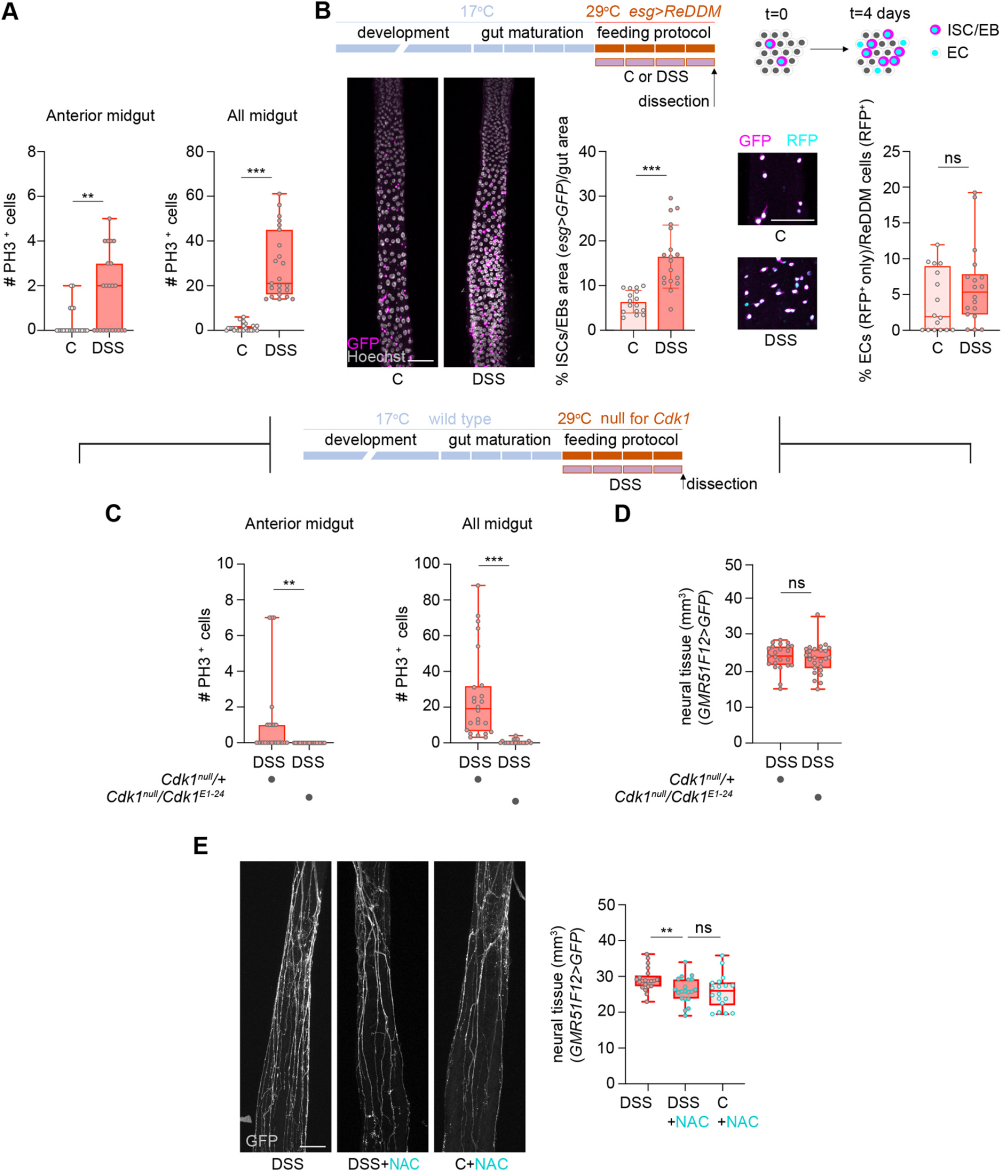
**Neural plasticity does not depend on intestinal stem cell proliferation and antioxidant treatment implicates reactive oxygen species as a contributor.** (A) Quantification of PH3/Hoechst^+^ cells in anterior and whole midgut of control and DSS-fed females. A red outline in the box and whisker plots indicates female. (B) Detailed experimental timeline schematic of ReDDM activation during feeding. Top rectangles represent 1 day each and reflect the age of the flies (blue and orange); bottom purple rectangles represent the duration of the feeding protocol. Confocal *z*-projections showing intestinal stem cells (ISCs)/enteroblasts (EBs) (magenta, GFP) and enterocytes (ECs) (cyan, RFP) in control and DSS-fed females, with quantification of ISC/EB area and the proportion of ECs. DSS-fed flies show a consistent, though not significant, increase in ECs. (C,D) Schematic of temperature-dependent *Cdk1* knockout (top); quantification of PH3/Hoechst^+^ cells (C) and neural tissue (D) in wild-type (heterozygous) and *Cdk1* null females after DSS feeding. (E) Confocal *z*-projections of neuronal processes in DSS, DSS+NAC and control+NAC-fed females with neural tissue quantification. Scale bars: 50 μm. **P<0.01, ***P<0.001; ns, not significant (Mann-Whitney test for A-C; ANCOVA for D,E).

To analyze the effect of gut damage on neural plasticity, we fed adult mated females DSS for 4 days and compared them to controls fed with the carrier (sucrose) (Fig. S1A-D). We confirmed the effectiveness of DSS by replicating previously published observations, including a reduction in lifespan (Amcheslavsky et al., 2009) (data not shown) and an increase in the proliferative response of ISCs in the midgut (Amcheslavsky et al., 2009) (see Fig. 2A). Over 90% of the flies that survived the feeding protocol tested negative for the SMURF assay (Rera et al., 2012), indicating that, in the majority of animals (93.3%±s.d.4.5) that were dissected, gut permeability and integrity were not compromised (Fig. S1E,F). Thus, during DSS feeding, neurons were largely shielded from direct exposure to gut contents that could potentially cause them damage. When we dissected the DSS-fed females and compared them to control flies, we observed a significant increase in neural tissue (Fig. 1B). Virgin flies also showed a DSS-dependent increase in neural tissue, comparable in magnitude to that observed in mated females (Fig. S1H,I). To rule out neurogenesis as the source of the new neural tissue, we quantified the number of neural nuclei in the HCG, the primary source of neurons innervating the anterior midgut (Fig. 1A). No differences were observed between control and DSS-fed animals (Fig. 1C), suggesting that the increase in neural tissue was due to neural growth. This neural growth was not exclusive to DSS damage, since we also observed it when feeding the flies bleomycin and H_2_O_2_ (Fig. 1D,E, Fig. S2A,B). In contrast, *Pseudomonas entomophila* infection led to a marked gut enlargement in our region of interest, with neural tissue scaling accordingly. Because this expansion likely approached the limit of neural growth, it prevented detection of any plasticity growth beyond this scaling response (Fig. S2C). Overall, several forms of chemically induced damage paradigms robustly trigger neural growth.

Tracheal terminal cells (TTCs) sprout in response to DSS, bleomycin, H_2_O_2_ and infectious pathogens (Medina et al., 2025; Perochon et al., 2021; Tamamouna et al., 2021), so we examined their behavior in the anterior midgut. This region showed sparse tracheation, which remained unchanged after DSS feeding, indicating that TTCs in this area were unresponsive to gut damage (Fig. S3A,B). Moreover, TTC morphology did not match the extent of neural growth, suggesting that they do not serve as a scaffold for neural tissue growth in this area.

We next explored the stability of this neural plasticity and conducted recovery experiments (Fig. 1F). While DSS feeding increased the neural tissue (C versus DSS), a 4-day recovery period (DSS+Rec) reverted the amount of neural tissue to levels as those of control flies (C+Rec). Consistent with this observation, a clear reversal of neural growth was observed when comparing flies dissected after the DSS treatment (DSS) and those dissected after the recovery phase (DSS+Rec). No differences were observed between control flies (C versus C+Rec).

In summary, these experiments indicate that the intact neural tissue innervating the digestive tube is plastic and responds to damage with growth. This neural growth is reversible after a recovery period, as the gut returns to homeostasis after injury. Importantly, these observations were validated with two independent neural drivers (*GMR51F12-GAL4*, Fig. 1 and *nSyb.S-GAL4*, Fig. S3C).

### ISC proliferation does not mediate neural plasticity

To determine whether ISC proliferation drove DSS-induced neural growth, we first confirmed that DSS feeding increased ISC division in the anterior midgut, as shown by elevated phospho-histone H3 (PH3) signal (Fig. 2A) and lineage tracing using ReDDM (Antonello et al., 2015) (Fig. 2B). To test causality, we blocked ISC cell division during DSS feeding using a combination of a *Cdk1* null allele (*Cdk1^null^*) and a temperature-sensitive allele (*Cdk^1E1–24^*) (Rodríguez et al., 2024) (Fig. 2C). Neural growth remained unaffected by the proliferation block (Fig. 2D), indicating that ISC division is not required for DSS-induced neural plasticity.

### Gut-derived reactive oxygen species are implicated neural plasticity

DSS feeding results in epithelial damage, causing cell death and an increase in reactive oxygen species (ROS) levels (Wu et al., 2012) (Fig. S4A). To test the contribution of ROS levels to neural growth, we co-fed flies with DSS and the ROS inhibitor N-acetyl cysteine (NAC). Neural growth was reduced in DSS+NAC flies and comparable to control+NAC levels (Fig. 2E), suggesting that DSS-dependent ROS contributes to neural plasticity. While this finding aligns with evidence that ROS supports structural plasticity (Oswald et al., 2018), it does not rule out additional gut-derived signals as potential regulators.

ROS from midgut cells induces Dh31 secretion from EEs for tracheal remodeling (Medina et al., 2025). Analysis of *Dh31* mutants and neuronal *Dh31-R* knockdowns showed normal baseline innervation and DSS-induced growth (Fig. S4D-G), consistent with plasticity proceeding independently of Dh31/Dh31-R signaling.

### Reversal of neural growth as the gut returns to homeostasis is not caspase dependent

We were curious about the mechanisms controlling neural growth reversal during recovery. Axon pruning can take place either through axon retraction or degeneration (Luo and O'Leary, 2005). During developmental remodeling, dendritic arborizing (da) sensory neurons ddaC degenerate (Williams and Truman, 2005) using local caspase activation to direct dendrite engulfment (Williams et al., 2006). To assess whether caspases mediated neural growth reversal, we inhibited apoptosis in neurons by overexpressing *Diap1*. Reversal still occurred (Fig. S5), suggesting that this process may result from axon retraction rather than caspase-dependent mechanisms.

### Neural plasticity is exclusive to females and is actively suppressed when female neurons are masculinized

**Fig. 3.**
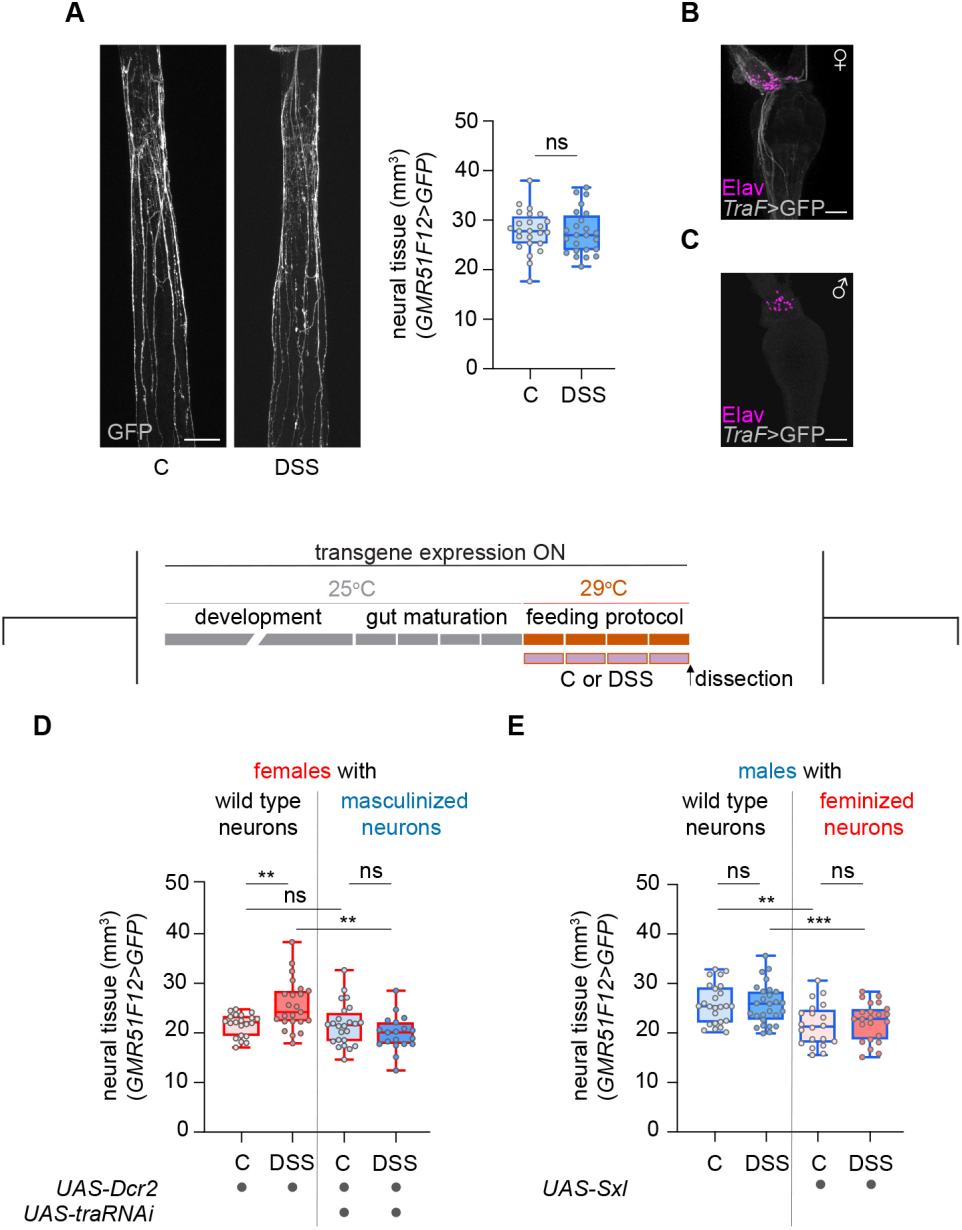
**Neural plasticity is not observed in males and masculinization of female neurons abolishes neural growth.** (A) Confocal *z*-projections and quantification of neural tissue in control and DSS-fed males (a blue outline in the box and whisker plots indicates males; a red outline indicates female). (B,C) HCG *z*-projections showing female-specific *tra* splicing (gray, GFP) in neurons (magenta, Elav) in females (B) and its absence in males (C). (D,E) Schematic of transgene expression and feeding protocol (top). Top rectangles represent 1 day each and reflect the age of the flies (gray and orange); bottom purple rectangles represent the duration of the feeding protocol. Quantification of neural tissue in females and in females with masculinized neurons (D), and in males and in males with feminized neurons (E) under control and DSS conditions. Scale bars: 50 μm. **P<0.01, ***P<0.001; ns, not significant (ANCOVA).

Interestingly, males did not exhibit neural growth following DSS treatment (Fig. 3A), despite a DSS-dependent increase in ROS (Fig. S4B) and reduced viability (Amcheslavsky et al., 2009). To investigate whether this neural growth was exclusive to female neurons, we aimed to create female individuals with masculinized neurons. Most sex differences in *Drosophila* arise from the female-specific expression of the *Sex lethal* gene (*Sxl*), which codes for an RNA-binding protein (Bell et al., 1988). Sxl induces the alternative splicing of the *transformer* (*tra*) gene, generating the female fate-determining TraF protein only in females (Belote et al., 1989; Boggs et al., 1987; Inoue et al., 1990; Sosnowski et al., 1989). We first confirmed that neurons in the HCG expressed the female-specific alternative splicing variant of *tra*, while males did not (Fig. 3B,C) (Hérault et al., 2024). Subsequently, we proceeded to masculinize neurons in females by means of *tra* downregulation with RNAi during development (Fig. 3D). We confirmed that the presence of the *UAS-Dcr2* transgene did not affect the response of wild-type females to DSS, and, thus, the expected increase in neural tissue was observed. Interestingly, DSS feeding did not have any effect on masculinized females, and the amount of neural tissue was not significantly different between control and DSS-fed flies. In addition, DSS-fed masculinized females displayed significantly less neural tissue than DSS-fed control flies, aligning with the absence of neural growth in masculinized females.

We also carried out the reverse experiment and attempted to confer plasticity to male neurons by feminizing them through developmental *Sxl* overexpression (Fig. 3E) (Bell et al., 1991). In this case, the DSS treatment had no effect on feminized males, and we did not observe the neural growth we expected to see for a successfully feminized male. Moreover, both control and DSS-fed feminized males showed less amount of neural tissue than wild-type males. These results indicate that neuronal Sxl overexpression in males is not sufficient to induce damage-responsive plasticity and may even impair neural tissue growth in our context.

These findings reveal a previously unrecognized sex-specific mechanism of neural plasticity in the fly gut, where female neurons exhibit a unique capacity for damage-induced growth that is actively suppressed by masculinization. In addition, the lack of neural growth in males following DSS-induced gut damage suggests that neural plasticity is limited in males compared to females.

### Female-specific neural plasticity impacts gut physiology and viability

**Fig. 4.**
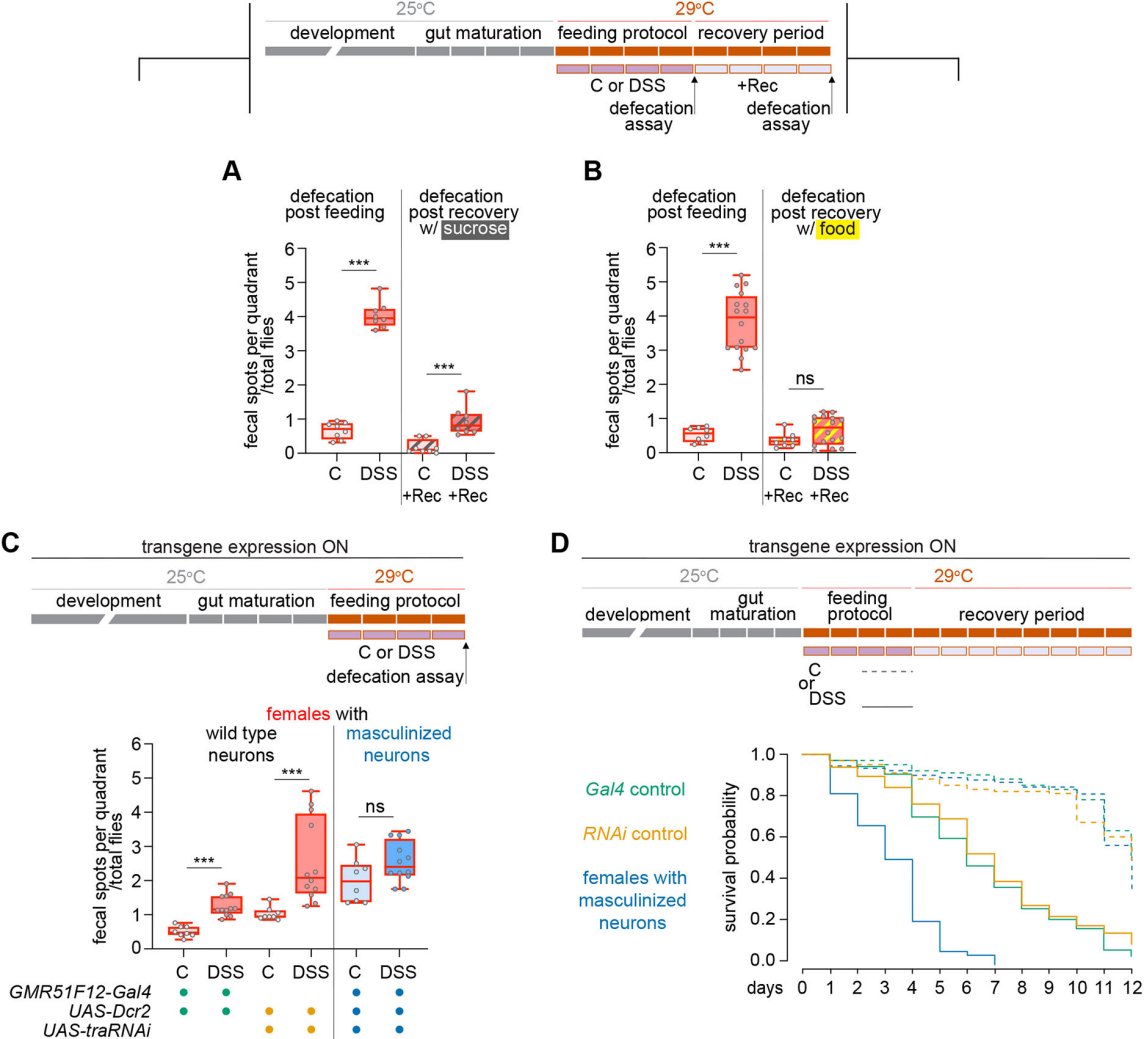
**Neural plasticity in females is required for the DSS-induced increase in defecation and it protects against DSS-induced mortality.** (A,B) Schematic shows the timing of defecation assays after control or DSS feeding and recovery on sucrose (A) or regular food (B), with corresponding quantifications. Top rectangles represent 1 day each and reflect the age of the flies (gray and orange); bottom purple rectangles represent the duration of the feeding protocol or any other additional period after that (light purple). A red outline in the box and whisker plots indicates female. (C) Schematic of transgene expression and assay timing (top); defecation after control or DSS feeding in females with wild-type (green, Gal4/dcr2 control; yellow, RNAi/dcr2 control) or masculinized neurons (blue). (D) Schematic of lifespan analysis covering feeding plus recovery (top); survival curves for same genotypes as in C. DSS-fed females with masculinized neurons show a significantly reduced lifespan (***P<0.001; log-rank test with Holm-Bonferroni correction). For additional comparisons, see Table S1. ***P<0.001; ns, not significant (Mann-Whitney test for A-C).

We assessed the impact of female-specific neural plasticity on gut function using defecation and survival assays. We tested defecation after DSS and recovery. DSS feeding strongly increased defecation (Fig. 4A,B). After recovery on sucrose, defecation remained high (Fig. 4A) despite neural tissue reverting (Fig. 1F), whereas recovery on standard food restored defecation to near-normal levels (Fig. 4B). This suggests that full normalization requires both gut and neural homeostasis.

To test if neural plasticity drives increased defecation and offers protection, we masculinized female neurons to block DSS-induced plasticity. This suppressed the defecation rise after DSS (Fig. 4C) and further reduced survival (Fig. 4D), showing that plasticity is required for both physiological response and viability.

### The *Drosophila* gut and its innervation as a model to study neural plasticity and sex-specific regulation

This system enables analysis of both growth and retraction phases of intact adult neurons after damage, capturing the dynamic nature of plasticity. We uncover a clear sexual dimorphism: plasticity occurs in females but is absent in males. In addition, plasticity is suppressed when female neurons are masculinized, while feminizing male neurons does not confer this suppression.

These findings open two complementary avenues for future investigation. First, sex-specific gene expression may underlie differential plasticity. Sex variations in ROS production or ROS-responsive pathways could drive female-specific growth, with candidate regulators including TOR and InR/PI3K/Akt, which are known to control regrowth in flies (Sanal et al., 2023; Yaniv and Schuldiner, 2016) and regeneration in mammals (Lund-Ricard et al., 2020). Also downstream of ROS, Unpaired (Upd) cytokines (Santabárbara-Ruiz et al., 2015) may also link tissue damage to neural remodeling. Second, surrounding tissues likely influence plasticity. The failure of feminized male neurons to grow suggests neuronal identity alone is insufficient – sex-specific cues from gut epithelium, visceral muscle, trachea or Malpighian tubules, such as secretion of Upd3 (Buchon et al., 2009a,b, 2010; Jiang et al., 2009; Lin et al., 2010; Liu et al., 2024; Medina et al., 2022; Osman et al., 2012; Tian et al., 2015), may be required. Upd ligands from these tissues could signal directly or indirectly to neurons (Malita et al., 2025; Sodders et al., 2025) to grow, while retraction could involve repulsive cues such as Ephrins, Slits or Semaphorins transiently expressed in tissues of the digestive tube environment, reminiscent of axon-guidance mechanisms (Luo and O'Leary, 2005). Together, the findings presented position the fly gut and its innervation as a model for dissecting how sex and tissue context shape neural plasticity, informing strategies for sex-specific neural repair.

## MATERIALS AND METHODS

### Fly stocks

The following fly lines were used: *GMR51F12-GAL4* (BDSC:58685), *nSyb.S-GAL4* (BDSC: 51635), *UAS-Flybow.1.1B* (used as 10xUAS-CD8::GFP, BDSC: 56802, 56803), *DSRF.Term-GAL4* (BDSC: 25753), *esg-GAL4* (a gift from M. Milán, unknown insertion, ICREA/IRB, Barcelona, Spain), *UAS-mCD8::GFP* (BDSC:5130), *UAS-H2B::RFP* (a gift from J. Morante, CSIC/Instituto de Neurociencias, Alicante, Spain), *tubP-GAL80^ts^* (BDSC: 7017), *Cdk1^null^* (BDSC: 6643), *Cdk^1E1–24^* (BDSC: 6641), *Dh31^attP^* (BDSC: 84490), *UAS-Dh31-R RNAi TRIPJF01945* (UAS-Dh31-R RNAi, BDSC: 25925), *Dh31-R^2A-Gal4^* (BDSC: 84626), *UAS-Diap1* (BDSC: 15310), *traF-GAL4* (a gift from B. Hudry, iBV, Nice, France), *UAS-Dcr-2* (BDSC: 24650), *UAS-tra RNAi TRiPJF03132* (*UAS-tra RNAi*, BDSC: 28512) and *UASp-Sxl.alt5-C8* (*UAS-Sxl*, BDSC: 58484). The complete list of genotypes is available in the supplementary Materials and Methods.

### Feeding protocols

For feeding assays, virgin females were collected and aged on standard media at 25°C for 3-4 days before undergoing mating for 24 h. The same procedure was carried out when experiments were performed with males. Flies were recovered from the mating and transferred in groups of 10 to an empty vial containing five pieces of 2 cm×2 cm of bench paper soaked with 500 ml of the desired feeding solution. Flies were transferred to a new vial with fresh feeding paper every day for the specified number of days prior to dissection. The feeding protocol was always carried out at 29°C, all feeding treatments were administered in 5% sucrose (S7903, Sigma Aldrich), and flies fed with sucrose alone served as the control (C) condition, unless otherwise stated. Flies were fed with DSS (42867, Sigma Aldrich) at 6% w/v for 4 days. For the SMURF assay, the last control and DSS feeding solutions before the day of dissection were laced with Brilliant Blue FCF (80717, Supelco) at 2.5% w/v. Bleomycin (BL7216, Sigma Aldrich) was administered at 50 µg ml^−1^ for 3 days, H_2_O_2_ (1072100250, Supelco) was administered at 1% for 4 days and *Pseudomonas entomophila* (*Pe*) was administered at an OD_600_=25 for 16 h. For NAC (A7250, Sigma Aldrich) co-treatment, flies were fed with 1,2 mM NAC in 5% sucrose (Control+NAC) or 6% DSS+1,2 mM NAC in 5% sucrose (DSS+NAC) for 4 days. For recovery (Rec) experiments, after the feeding protocol, C and DSS-fed flies were each transferred to vials with 5% sucrose for 4 days (C+Rec; DSS+Rec). To carry out comparisons within the same cohort of flies, a subset of flies was dissected after the feeding protocol, and the rest after the recovery period.

### Temperature-sensitive assays

To visualize the amount of proliferation taking place exclusively during the 4 days of the feeding protocol, we set ReDDM-based crosses (Antonello et al., 2015). This lineage-tracing approach uses the Gal80 temperature-sensitive allele (*Gal80^ts^*), which inhibits GAL4 transcription at permissive temperature (19°C) and allows it at the restrictive one (29°C) (McGuire et al., 2003), and *esg-GAL4* to label ISCs and EBs. Crosses were grown and flies were kept at 17°C to ensure Gal80^ts^ repression until adult mated female flies were shifted to 29°C to inactivate Gal80 and carry out the feeding protocol. To block ISC proliferation during the feeding protocol, *Cdk1^null^/ Cdk^1E1–24^* flies were grown, aged and mated at 17°C, Cdk1 function was abrogated once the flies were transferred to 29°C (Rodriguez et al., 2024) during the DSS feeding protocol.

### Dissection and immunostaining

To obtain the gut, flies were dissected in PBS. Adult animals had their heads cut off with scissors by the neck, and their abdomens were pulled apart from the thorax and opened to extract the gut. With this procedure, the digestive tube was severed well above the crop duct. Once the fat and ovaries were removed, the gut was handled by its posterior end and transferred to PBS in a well drawn onto a poly-L-lysine-coated slide (P1524, Merck) using hydrophobic silicone (Flowsil, Intek Adhesives). In cases where the anterior part of the midgut had to be repositioned, we manipulated the gut from the crop to avoid touching the digestive tube. Guts were fixed at room temperature for 30 min in 4% PFA in PBS. Washes were carried out with PBS-T (PBS, 0.25% Triton X-100). To label the muscle or nuclei, guts were incubated at room temperature for 30 min with Alexa Fluor 635 Phalloidin (1/400, A34054, Invitrogen) or Hoechst 33258 (1/1000, H3569, Invitrogen), respectively, in PBS-T. A final wash with PBS was carried out before mounting. When antibodies were used, primaries were incubated overnight at 4°C and secondaries were incubated at room temperature for 2-3 h. The following antibodies were used: rat-anti-Elav (1/100, 7E8A10, DSHB), mouse-anti-Discs large (1/50, 4F3, DSHB), rabbit-anti-PH3 (1/1000, 06-570, EMD Millipore), rabbit-anti-DsRed (1/200, 632496, Clontech), chicken-anti-GFP (1/800, ab13970, Abcam), and Alexa Fluor 568 goat-anti-rat, Alexa Fluor 488 goat-anti-mouse, Alexa Fluor 488 goat-anti-rabbit, Alexa Fluor 568 goat-anti-rabbit, Alexa Fluor 488 goat-anti-chicken (1/500; A11077, A11001, A11008, A11011, A11039, respectively; Life Technologies). Samples were mounted in VECTASHIELD PLUS Antifade Mounting (H-1900-10, Vector Laboratories).

### ROS detection

C and DSS-fed guts were dissected in Schneider's medium (50146, Sigma Aldrich) and placed on poly-L-lysine-coated slides. Tissues were incubated in CM-H_2_DCFDA (C6827, Invitrogen) at 2 µM in Schneider's medium for 30 min in the dark, then washed three times in Schneider's medium before imaging. Dissection time was limited to 15 min for each experimental group, and imaging was performed within 45 min of dissection.

### Defecation assay

Flies under analysis were starved for 1 h in empty vials. Subsequently, 20-25 flies were introduced into fly cages positioned over a 60-mm Petri dish containing a central 5×5 mm piece of grape-juice agar with 2.5% (w/v) Brilliant Blue FCF (Supelco, 80717). Flies were kept at 29°C for 12 h in constant darkness before quantification of the fecal spots.

### Lifespan experiments

Lifespan analysis of females aged and mated started on the 1st day of the feeding protocol and after that continued for 8 additional days on 5% sucrose. Flies were placed in groups of maximum 10 per vial and transferred to new feeding vials every day.

### Microscopy

Samples were imaged using a Zeiss LSM 880 confocal microscope equipped with a 25×0.8 glycerol immersion media objective and an Argon and a HeNe633 lasers. For neural network analysis, to consistently image the same region in each gut, we used the end of the proventriculus as a positional landmark and captured the gut section that fitted within the visual field of the 25× objective at 0.8 magnification, which in our setup generated images of 566.79 µm^2^. As quality control for dissection, only guts with an intact HCG were considered since this ganglion contains the cell bodies of many of the neurons innervating the anterior midgut. Stacks of images were acquired with a pixel size of 0.55×0.55×1 μm (*xyz*) and a pixel dwell time of 2.05 μs.

### Image processing and quantification based on images

Images were processed with Fiji (version ImageJ 1.53c; Schindelin et al., 2012) and figures assembled using Adobe Illustrator.

#### Quantification of neural tissue and gut parameters

The channel with the neuron staining was filtered in 3D (Ollion et al., 2013) first with the maximum filter and then with the minimum filter, with a radius of 3.3 μm in *x* and *y* and 2 μm in *z*, in both filters. The filtered stack was then processed using the Tubeness plug-in (sigma=0.66) (Sato et al., 1998). Finally, images were binarized using the Bernsen's AutoLocal threshold method (radius=2) (Bernsen, 1986). The resulting binary stack was analyzed first with the 3D Object Counter function to obtain the total neuronal network volume (mm^3^). The channel with the gut staining was processed by background subtraction and then thresholded using the Li's method (Li and Lee, 1993). The resulting binary stack was analyzed with the 3D Object Counter function to obtain the volume of the gut.

#### Other quantifications on images

The number of Elav/Hoechst-positive nuclei in the HCG was counted from the confocal stack using the 3D Object Counter plug-in. The number of TTC nuclei was counted by hand from the confocal stack maximum projection. TTC volume was obtained using the same procedure as for neural tissue quantification. The number of PH3/Hoechst-positive nuclei in the midgut was counted directly under the confocal microscope. For quantification of cumulative proliferation using ReDDM, *xyz* stacks of half the gut were taken and *z*-projected. The Hoechst signal was manually outlined to measure the gut area. To quantify the area of GFP signal from mCD8::GFP-labeled cells (representing ISCs and EBs resulting from ISCs divisions) a Gaussian Blur filter (radius=2.0) was applied. The image was then thresholded using the ‘MaxEntropy’ algorithm (Kapur et al., 1985), and, finally, the GFP-positive regions larger than 10 µm^2^ were quantified. The GFP area was represented as a percentage of the total area. To quantify the number of differentiated ECs, the total number of ReDDM cells was quantified manually using the H2B::RFP nuclear signal and the presence or absence of cytoplasmic mCD8::GFP was tracked to calculate the percentage of differentiated ECs. For ROS signal analysis, the same region used for neural network evaluation was imaged. *Z*-projections combining 15 optical sections were generated using maximum intensity to capture the epithelial signal. For each gut image, mean pixel intensity was quantified in two distinct areas to account for variability across the imaged gut region.

### Other quantifications

*SMURF assay*: on the 4th day of the DSS feeding protocol, we assessed the proportion of surviving flies that were positive for the SMURF phenotype. Ten tubes with 10 flies each were used in each of three biologically independent replicates.

For the defecation assay, the Petri dish was removed after incubation, and the four quadrants of the dish were photographed separately under a stereomicroscope. Blue fecal spots in each quadrant were manually counted using ImageJ and normalized by the total number of flies in the cage. Thus, the value obtained represented fecal spots per quadrant/total flies in the cage. Each quadrant was treated as an independent technical replicate. This approach increased the number of measurements per biological replicate, thereby improving the statistical power of the analysis while ensuring complete and accurate counting of all fecal deposits. Flies from the same cohort were used in experiments where defecation assays were carried out after DSS treatment and after recovery treatment. For lifespan experiments, dead flies were counted every 24 h before being transferred to a new feeding vial.

### Statistics

Data analysis was carried out using Prism 6 (GraphPad Software) and Statgraphics Centurion version 18 (Statgraphics Technologies). Table S1 provides statistical details (sample size for each condition, definition of values, statistical test, comparisons and *P* values) for each main and supplementary figure. All tests are two-tailed. For data shown in box plots, the median is given between the first and third quartiles (ends of the box). Whiskers represent the maximum and minimum values of the data. Bar graphs display the mean, with error bars representing the standard deviation.

To analyze the effect of the treatment on neural tissue volume and TTCs tissue volume, analysis of covariance (ANCOVA) was performed. The dependent variable was neural tissue volume or TTCs tissue volume, the fixed factor was the treatment and the covariate was gut volume (Fig. 1B,D-F; Fig. 2D,E; Fig. 3A,D,E; Fig. S1A-D,G-H; Fig. S2A-C; Fig. S3B,C; Fig. S4D-G; Fig. S5).

For pairwise comparisons of data sets following a normal distribution (Fig. 1C, Fig. S4A,B), an unpaired *t*-test was performed. For pairwise comparisons of data sets not following a normal distribution, we used the non-parametric Mann–Whitney test (Fig. 2A-C; Fig. 4A-C; Fig. S3A).

For lifespan experiments, survival distributions were compared using the non-parametric log-rank test (Kaplan and Meier, 1958), which accounts for censored data. To correct for multiple comparisons, the Holm–Bonferroni method was applied to control the family-wise error rate (Holm, 1979).

## Supplementary Material

10.1242/develop.204945_sup1 Supplementary information

Table S1. Statistical details for Figures and Supplementary figures

## References

[DEV204945C1] Amcheslavsky, A., Jiang, J. and Ip, Y. T. (2009). Tissue damage-induced intestinal stem cell division in Drosophila. *Cell Stem Cell* 4, 49-61. 10.1016/j.stem.2008.10.016 19128792 PMC2659574

[DEV204945C82] Ameku, T., Beckwith, H., Blackie, L. and Miguel-Aliaga, I. (2020). Food, microbes, sex and old age: on the plasticity of gastrointestinal innervation. *Curr. Opin. Neurobiol.* 62, 83-91. 10.1016/j.conb.2019.12.004 32028080 PMC7294223

[DEV204945C2] Antonello, Z. A., Reiff, T., Ballesta-Illan, E. and Dominguez, M. (2015). Robust intestinal homeostasis relies on cellular plasticity in enteroblasts mediated by miR-8–Escargot switch. *EMBO J.* 34, 2025-2041. 10.15252/embj.201591517 26077448 PMC4551350

[DEV204945C3] Bell, L. R., Maine, E. M., Schedl, P. and Cline, T. W. (1988). Sex-lethal, a Drosophila sex determination switch gene, exhibits sex-specific RNA splicing and sequence similarity to RNA binding proteins. *Cell* 55, 1037-1046. 10.1016/0092-8674(88)90248-6 3144435

[DEV204945C4] Bell, L. R., Horabin, J. I., Schedl, P. and Cline, T. W. (1991). Positive autoregulation of Sex-lethal by alternative splicing maintains the female determined state in Drosophila. *Cell* 65, 229-239. 10.1016/0092-8674(91)90157-T 2015624

[DEV204945C5] Belote, J. M., McKeown, M., Boggs, R. T., Ohkawa, R. and Sosnowski, B. A. (1989). Molecular genetics of *transformer*, a genetic switch controlling sexual differentiation in *Drosophila*. *Dev. Genet.* 10, 143-154. 10.1002/dvg.1020100304 2472240

[DEV204945C6] Bentley, D. and Toroian-Raymond, A. (1981). Embryonic and postembryonic morphogenesis of a grasshopper interneuron. *J. Comp. Neurol.* 201, 507-518. 10.1002/cne.902010404 7287932

[DEV204945C7] Bernsen, J. (1986). Dynamic thresholding of gray level image. *Proc. Int. Conf. Pattern Recognit. Berl. ICPR* 86, 1251-1255.

[DEV204945C8] Boggs, R. T., Gregor, P., Idriss, S., Belote, J. M. and McKeown, M. (1987). Regulation of sexual differentiation in D. melanogaster via alternative splicing of RNA from the transformer gene. *Cell* 50, 739-747. 10.1016/0092-8674(87)90332-1 2441872

[DEV204945C9] Bucher, D. and Pflüger, H.-J. (2000). Directional sensitivity of an identified wind-sensitive interneuron during the postembryonic development of the locust. *J. Insect Physiol.* 46, 1545-1556. 10.1016/S0022-1910(00)00078-0 10980300

[DEV204945C10] Buchon, N., Broderick, N. A., Chakrabarti, S. and Lemaitre, B. (2009a). Invasive and indigenous microbiota impact intestinal stem cell activity through multiple pathways in *Drosophila*. *Genes Dev.* 23, 2333-2344. 10.1101/gad.1827009 19797770 PMC2758745

[DEV204945C11] Buchon, N., Broderick, N. A., Poidevin, M., Pradervand, S. and Lemaitre, B. (2009b). Drosophila intestinal response to bacterial infection: activation of host defense and stem cell proliferation. *Cell Host Microbe* 5, 200-211. 10.1016/j.chom.2009.01.003 19218090

[DEV204945C12] Buchon, N., Broderick, N. A., Kuraishi, T. and Lemaitre, B. (2010). DrosophilaEGFR pathway coordinates stem cell proliferation and gut remodeling following infection. *BMC Biol.* 8, 152. 10.1186/1741-7007-8-152 21176204 PMC3022776

[DEV204945C13] Buchon, N., Osman, D., David, F. P. A., Yu Fang, H., Boquete, J.-P., Deplancke, B. and Lemaitre, B. (2013). Morphological and molecular characterization of adult midgut compartmentalization in Drosophila. *Cell Rep.* 3, 1725-1738. 10.1016/j.celrep.2013.04.001 23643535

[DEV204945C14] Chen, J., Reiher, W., Hermann-Luibl, C., Sellami, A., Cognigni, P., Kondo, S., Helfrich-Förster, C., Veenstra, J. A. and Wegener, C. (2016). Allatostatin A signalling in Drosophila regulates feeding and sleep and is modulated by PDF. *PLoS Genet.* 12, e1006346. 10.1371/journal.pgen.1006346 27689358 PMC5045179

[DEV204945C15] Cirillo, C., Brihmat, N., Castel-Lacanal, E., Le Friec, A., Barbieux-Guillot, M., Raposo, N., Pariente, J., Viguier, A., Simonetta-Moreau, M., Albucher, J.-F. et al. (2020). Post-stroke remodeling processes in animal models and humans. *J. Cereb. Blood Flow Metab.* 40, 3-22. 10.1177/0271678X19882788 31645178 PMC6928555

[DEV204945C16] Cognigni, P., Bailey, A. P. and Miguel-Aliaga, I. (2011). Enteric neurons and systemic signals couple nutritional and reproductive status with intestinal homeostasis. *Cell Metab.* 13, 92-104. 10.1016/j.cmet.2010.12.010 21195352 PMC3038267

[DEV204945C17] Cui, X., Meiselman, M. R., Thornton, S. N. and Yapici, N. (2024). A gut-brain-gut interoceptive circuit loop gates sugar ingestion in Drosophila. *bioRxiv*. 10.1101/2024.09.02.610892

[DEV204945C18] Gao, Z., Pang, Z., Chen, Y., Lei, G., Zhu, S., Li, G., Shen, Y. and Xu, W. (2022). Restoring after central nervous system injuries: neural mechanisms and translational applications of motor recovery. *Neurosci. Bull.* 38, 1569-1587. 10.1007/s12264-022-00959-x 36333482 PMC9723055

[DEV204945C83] Hadjieconomou, D., King, G., Gaspar, P., Mineo, A., Blackie, L., Ameku, T., Studd, C., De Mendoza, A., Diao, F., White, B. H. et al. (2020). Enteric neurons increase maternal food intake during reproduction. *Nature* 587, 455-459. 10.1038/s41586-020-2866-8 33116314 PMC7610780

[DEV204945C19] Hérault, C., Pihl, T. and Hudry, B. (2024). Cellular sex throughout the organism underlies somatic sexual differentiation. *Nat. Commun.* 15, 6925. 10.1038/s41467-024-51228-6 39138201 PMC11322332

[DEV204945C20] Holm, S. (1979). A simple sequentially rejective multiple test procedure. *Scand. J. Stat.* 6, 65-70.

[DEV204945C84] Holsopple, J. M., Cook, K. R. and Popodi, E. M. (2022). Enteroendocrine cell expression of split-GAL4 drivers bearing regulatory sequences associated with panneuronally expressed genes in *Drosophila melanogaster*. *MicroPubl. Biol.* 10.17912/micropub.biology.000628 PMC9440388 36065255

[DEV204945C22] Howard, A. M., LaFever, K. S., Fenix, A. M., Scurrah, C. R., Lau, K. S., Burnette, D. T., Bhave, G., Ferrell, N. and Page-McCaw, A. (2019). DSS-induced damage to basement membranes is repaired by matrix replacement and crosslinking. *J. Cell Sci.* 132, jcs226860. 10.1242/jcs.226860 30837285 PMC6467488

[DEV204945C23] Hyer, M. M., Phillips, L. L. and Neigh, G. N. (2018). Sex differences in synaptic plasticity: hormones and beyond. *Front. Mol. Neurosci.* 11, 266. 10.3389/fnmol.2018.00266 30108482 PMC6079238

[DEV204945C24] Inoue, K., Hoshijima, K., Sakamoto, H. and Shimura, Y. (1990). Binding of the Drosophila Sex-lethal gene product to the alternative splice site of transformer primary transcript. *Nature* 344, 461-463. 10.1038/344461a0 1690860

[DEV204945C25] Jenett, A., Rubin, G. M., Ngo, T.-T. B., Shepherd, D., Murphy, C., Dionne, H., Pfeiffer, B. D., Cavallaro, A., Hall, D., Jeter, J. et al. (2012). A GAL4-Driver Line Resource for Drosophila Neurobiology. *Cell Rep.* 2, 991-1001. 10.1016/j.celrep.2012.09.011 23063364 PMC3515021

[DEV204945C26] Jiang, H., Patel, P. H., Kohlmaier, A., Grenley, M. O., McEwen, D. G. and Edgar, B. A. (2009). Cytokine/Jak/Stat signaling mediates regeneration and homeostasis in the Drosophila midgut. *Cell* 137, 1343-1355. 10.1016/j.cell.2009.05.014 19563763 PMC2753793

[DEV204945C27] Jiang, H., Tian, A. and Jiang, J. (2016). Intestinal stem cell response to injury: lessons from Drosophila. *Cell. Mol. Life Sci.* 73, 3337-3349. 10.1007/s00018-016-2235-9 27137186 PMC4998060

[DEV204945C28] Kaplan, E. L. and Meier, P. (1958). Nonparametric estimation from incomplete observations. *J. Am. Stat. Assoc.* 53, 457-481. 10.1080/01621459.1958.10501452

[DEV204945C29] Kapur, J. N., Sahoo, P. K. and Wong, A. K. C. (1985). A new method for gray-level picture thresholding using the entropy of the histogram. *Comput. Vis. Graph. Image Process.* 29, 273-285. 10.1016/0734-189X(85)90125-2

[DEV204945C30] Kenmoku, H., Ishikawa, H., Ote, M., Kuraishi, T. and Kurata, S. (2016). A subset of neurons controls the permeability of the peritrophic matrix and midgut structure in *Drosophila* adults. *J. Exp. Biol.* 30, jeb.122960. 10.1242/jeb.122960 27229474

[DEV204945C31] Kirby, E. D., Andrushko, J. W., Rinat, S., D'Arcy, R. C. N. and Boyd, L. A. (2024). Investigating female versus male differences in white matter neuroplasticity associated with complex visuo-motor learning. *Sci. Rep.* 14, 5951. 10.1038/s41598-024-56453-z 38467763 PMC10928090

[DEV204945C32] Ksiazek-Winiarek, D. J., Szpakowski, P. and Glabinski, A. (2015). Neural plasticity in multiple sclerosis: the functional and molecular background. *Neural Plast.* 2015, 1-11. 10.1155/2015/307175 PMC4503575 26229689

[DEV204945C33] Kuraishi, T., Kenmoku, H. and Kurata, S. (2015). From mouth to anus: functional and structural relevance of enteric neurons in the Drosophila melanogaster gut. *Insect Biochem. Mol. Biol.* 67, 21-26. 10.1016/j.ibmb.2015.07.003 26232723

[DEV204945C34] Lee, S. and Stevens, C. F. (2007). General design principle for scalable neural circuits in a vertebrate retina. *Proc. Natl. Acad. Sci. USA* 104, 12931-12935. 10.1073/pnas.0705469104 17646664 PMC1931479

[DEV204945C35] Lemaitre, B. and Miguel-Aliaga, I. (2013). The digestive tract of *Drosophila melanogaster*. *Annu. Rev. Genet.* 47, 377-404. 10.1146/annurev-genet-111212-133343 24016187

[DEV204945C36] Li, C. H. and Lee, C. K. (1993). Minimum cross entropy thresholding. *Pattern Recognit.* 26, 617-625. 10.1016/0031-3203(93)90115-D

[DEV204945C37] Lin, G., Xu, N. and Xi, R. (2010). Paracrine unpaired signaling through the JAK/STAT pathway controls self-renewal and lineage differentiation of drosophila intestinal stem cells. *J. Mol. Cell Biol.* 2, 37-49. 10.1093/jmcb/mjp028 19797317

[DEV204945C38] Liu, Z., Zhang, H., Lemaitre, B. and Li, X. (2024). Duox activation in Drosophila Malpighian tubules stimulates intestinal epithelial renewal through a countercurrent flow. *Cell Rep.* 43, 114109. 10.1016/j.celrep.2024.114109 38613782

[DEV204945C39] Lund-Ricard, Y., Cormier, P., Morales, J. and Boutet, A. (2020). mTOR signaling at the crossroad between metazoan regeneration and human diseases. *Int. J. Mol. Sci.* 21, 2718. 10.3390/ijms21082718 32295297 PMC7216262

[DEV204945C40] Luo, L. and O'Leary, D. D. M. (2005). Axon retraction and degeneration in development and disease. *Annu. Rev. Neurosci.* 28, 127-156. 10.1146/annurev.neuro.28.061604.135632 16022592

[DEV204945C41] Mahar, M. and Cavalli, V. (2018). Intrinsic mechanisms of neuronal axon regeneration. *Nat. Rev. Neurosci.* 19, 323-337. 10.1038/s41583-018-0001-8 29666508 PMC5987780

[DEV204945C42] Malita, A., Skakkebaek, A. H., Kubrak, O., Chen, X., Koyama, T., Connolly, E. C., Ahrentloev, N., Andersen, D. S., Texada, M. J., Halberg, K. et al. (2025). Glia-mediated gut–brain cytokine signaling couples sleep to intestinal inflammatory responses induced by oxidative stress. *eLife* 13, RP99999. 10.7554/eLife.99999 40924804 PMC12419797

[DEV204945C43] Marianes, A. and Spradling, A. C. (2013). Physiological and stem cell compartmentalization within the Drosophila midgut. *eLife* 2, e00886. 10.7554/eLife.00886 23991285 PMC3755342

[DEV204945C44] McGuire, S. E., Le, P. T., Osborn, A. J., Matsumoto, K. and Davis, R. L. (2003). Spatiotemporal rescue of memory dysfunction in *Drosophila*. *Science* 302, 1765-1768. 10.1126/science.1089035 14657498

[DEV204945C45] Medina, A., Bellec, K., Polcowñuk, S. and Cordero, J. B. (2022). Investigating local and systemic intestinal signalling in health and disease with *Drosophila*. *Dis. Model. Mech.* 15, dmm049332. 10.1242/dmm.049332 35344037 PMC8990086

[DEV204945C46] Medina, A. B., Perochon, J., Tian, Y., Johnson, C. T., Holcombe, J., Ramesh, P., Polcowñuk, S., Yu, Y. and Cordero, J. B. (2025). Neuroendocrine control of intestinal regeneration through the vascular niche in Drosophila. *Dev. Cell* 60, 3085-3101.e6. 10.1016/j.devcel.2025.06.036 40695286

[DEV204945C47] Menon, K. P., Carrillo, R. A. and Zinn, K. (2013). Development and plasticity of the *Drosophila* larval neuromuscular junction. *WIREs Dev. Biol.* 2, 647-670. 10.1002/wdev.108 PMC3767937 24014452

[DEV204945C48] Miguel-Aliaga, I., Jasper, H. and Lemaitre, B. (2018). Anatomy and physiology of the digestive tract of *Drosophila melanogaster*. *Genetics* 210, 357-396. 10.1534/genetics.118.300224 30287514 PMC6216580

[DEV204945C49] Min, S., Oh, Y., Verma, P., Whitehead, S. C., Yapici, N., Van Vactor, D., Suh, G. S. B. and Liberles, S. (2021). Control of feeding by Piezo-mediated gut mechanosensation in Drosophila. *eLife* 10, e63049. 10.7554/eLife.63049 33599608 PMC7920550

[DEV204945C50] Murphy, T. H. and Corbett, D. (2009). Plasticity during stroke recovery: from synapse to behaviour. *Nat. Rev. Neurosci.* 10, 861-872. 10.1038/nrn2735 19888284

[DEV204945C51] Nudo, R. J. (2003). Functional and structural plasticity in motor cortex: implications for stroke recovery. *Phys. Med. Rehabil. Clin. N. Am.* 14, S57-S76. 10.1016/S1047-9651(02)00054-2 12625638

[DEV204945C52] Olds, W. H. and Xu, T. (2014). Regulation of food intake by mechanosensory ion channels in enteric neurons. *eLife* 3, e04402. 10.7554/eLife.04402 25285450 PMC4225495

[DEV204945C53] Ollion, J., Cochennec, J., Loll, F., Escudé, C. and Boudier, T. (2013). TANGO: a generic tool for high-throughput 3D image analysis for studying nuclear organization. *Bioinformatics* 29, 1840-1841. 10.1093/bioinformatics/btt276 23681123 PMC3702251

[DEV204945C54] Osman, D., Buchon, N., Chakrabarti, S., Huang, Y.-T., Su, W.-C., Poidevin, M., Tsai, Y.-C. and Lemaitre, B. (2012). Autocrine and paracrine unpaired signaling regulate intestinal stem cell maintenance and division. *J. Cell Sci.* 125, 5944-5949. 10.1242/jcs.113100 23038775

[DEV204945C55] Oswald, M. C. W., Garnham, N., Sweeney, S. T. and Landgraf, M. (2018). Regulation of neuronal development and function by ROS. *FEBS Lett.* 592, 679-691. 10.1002/1873-3468.12972 29323696 PMC5888200

[DEV204945C56] Perochon, J., Yu, Y., Aughey, G. N., Medina, A. B., Southall, T. D. and Cordero, J. B. (2021). Dynamic adult tracheal plasticity drives stem cell adaptation to changes in intestinal homeostasis in Drosophila. *Nat. Cell Biol.* 23, 485-496. 10.1038/s41556-021-00676-z 33972729 PMC7610788

[DEV204945C57] Petersen, E. D., Sharkey, E. D., Pal, A., Shafau, L. O., Zenchak-Petersen, J., Peña, A. J., Aggarwal, A., Prakash, M. and Hochgeschwender, U. (2022). Restoring function after severe spinal cord injury through BioLuminescent-OptoGenetics. *Front. Neurol.* 12, 792643. 10.3389/fneur.2021.792643 35126293 PMC8811305

[DEV204945C58] Petsakou, A., Liu, Y., Liu, Y., Comjean, A., Hu, Y. and Perrimon, N. (2023). Cholinergic neurons trigger epithelial Ca2+ currents to heal the gut. *Nature* 623, 122-131. 10.1038/s41586-023-06627-y 37722602 PMC10699467

[DEV204945C59] Prosperini, L., Piattella, M. C., Giannì, C. and Pantano, P. (2015). Functional and structural brain plasticity enhanced by motor and cognitive rehabilitation in multiple sclerosis. *Neural Plast.* 2015, 1-12. 10.1155/2015/481574 PMC4438192 26064692

[DEV204945C60] Rera, M., Clark, R. I. and Walker, D. W. (2012). Intestinal barrier dysfunction links metabolic and inflammatory markers of aging to death in Drosophila. *Proc. Natl. Acad. Sci. USA* 109, 21528-21533. 10.1073/pnas.1215849110 23236133 PMC3535647

[DEV204945C61] Rodríguez, A., Foronda, D., Córdoba, S., Felipe-Cordero, D., Baonza, A., Miguez, D. G. and Estella, C. (2024). Cell proliferation and Notch signaling coordinate the formation of epithelial folds in the *Drosophila* leg. *Development* 151, dev202384. 10.1242/dev.202384 38512712 PMC11058088

[DEV204945C62] Sampaio-Baptista, C., Sanders, Z.-B. and Johansen-Berg, H. (2018). Structural plasticity in adulthood with motor learning and stroke rehabilitation. *Annu. Rev. Neurosci.* 41, 25-40. 10.1146/annurev-neuro-080317-062015 29490196

[DEV204945C63] Sanal, N., Keding, L., Gigengack, U., Michalke, E. and Rumpf, S. (2023). TORC1 regulation of dendrite regrowth after pruning is linked to actin and exocytosis. *PLoS Genet.* 19, e1010526. 10.1371/journal.pgen.1010526 37167328 PMC10204957

[DEV204945C64] Santabárbara-Ruiz, P., López-Santillán, M., Martínez-Rodríguez, I., Binagui-Casas, A., Pérez, L., Milán, M., Corominas, M. and Serras, F. (2015). ROS-induced JNK and p38 signaling is required for unpaired cytokine activation during Drosophila regeneration. *PLoS Genet.* 11, e1005595. 10.1371/journal.pgen.1005595 26496642 PMC4619769

[DEV204945C65] Sato, Y., Nakajima, S., Shiraga, N., Atsumi, H., Yoshida, S., Koller, T., Gerig, G. and Kikinis, R. (1998). Three-dimensional multi-scale line filter for segmentation and visualization of curvilinear structures in medical images. *Med. Image Anal.* 2, 143-168. 10.1016/S1361-8415(98)80009-1 10646760

[DEV204945C66] Schindelin, J., Arganda-Carreras, I., Frise, E., Kaynig, V., Longair, M., Pietzsch, T., Preibisch, S., Rueden, C., Saalfeld, S., Schmid, B. et al. (2012). Fiji: an open-source platform for biological-image analysis. *Nat. Methods* 9, 676-682. 10.1038/nmeth.2019 22743772 PMC3855844

[DEV204945C67] Sodders, M., Das, A. and Bai, H. (2025). Glial peroxisome dysfunction induces axonal swelling and neuroinflammation in *Drosophila*. *G3* 15, jkae243. 10.1093/g3journal/jkae243 39385706 PMC11708211

[DEV204945C68] Sosnowski, B. A., Belote, J. M. and McKeown, M. (1989). Sex-specific alternative splicing of RNA from the transformer gene results from sequence-dependent splice site blockage. *Cell* 58, 449-459. 10.1016/0092-8674(89)90426-1 2503251

[DEV204945C69] Tamamouna, V., Rahman, M. M., Petersson, M., Charalambous, I., Kux, K., Mainor, H., Bolender, V., Isbilir, B., Edgar, B. A. and Pitsouli, C. (2021). Remodelling of oxygen-transporting tracheoles drives intestinal regeneration and tumorigenesis in Drosophila. *Nat. Cell Biol.* 23, 497-510. 10.1038/s41556-021-00674-1 33972730 PMC8567841

[DEV204945C70] Tedeschi, A. and Bradke, F. (2017). Spatial and temporal arrangement of neuronal intrinsic and extrinsic mechanisms controlling axon regeneration. *Curr. Opin. Neurobiol.* 42, 118-127. 10.1016/j.conb.2016.12.005 28039763

[DEV204945C71] Tian, A., Shi, Q., Jiang, A., Li, S., Wang, B. and Jiang, J. (2015). Injury-stimulated Hedgehog signaling promotes regenerative proliferation of *Drosophila* intestinal stem cells. *J. Cell Biol.* 208, 807-819. 10.1083/jcb.201409025 25753035 PMC4362464

[DEV204945C72] Tian, A., Morejon, V., Kohoutek, S., Huang, Y. C., Deng, W. M. and Jiang, J. (2022). Damage-induced regeneration of the intestinal stem cell pool through enteroblast mitosis in the *Drosophila* midgut. *EMBO J.* 41, e110834. 10.15252/embj.2022110834 35950466 PMC9531297

[DEV204945C73] Titos, I., Juginović, A., Vaccaro, A., Nambara, K., Gorelik, P., Mazor, O. and Rogulja, D. (2023). A gut-secreted peptide suppresses arousability from sleep. *Cell* 186, 1382-1397.e21. 10.1016/j.cell.2023.02.022 36958331 PMC10216829

[DEV204945C74] Turtzo, L. C. and McCullough, L. D. (2008). Sex differences in stroke. *Cerebrovasc. Dis.* 26, 462-474. 10.1159/000155983 18810232 PMC2637395

[DEV204945C75] Weaver, L. N., Ma, T. and Drummond-Barbosa, D. (2020). Analysis of Gal4 expression patterns in adult *Drosophila* females. *G3* 10, 4147-4158. 10.1534/g3.120.401676 32917721 PMC7642949

[DEV204945C76] Williams, D. W. and Truman, J. W. (2005). Cellular mechanisms of dendrite pruning in *Drosophila*: insights from in vivo time-lapse of remodeling dendritic arborizing sensory neurons. *Development* 132, 3631-3642. 10.1242/dev.01928 16033801

[DEV204945C77] Williams, D. W., Kondo, S., Krzyzanowska, A., Hiromi, Y. and Truman, J. W. (2006). Local caspase activity directs engulfment of dendrites during pruning. *Nat. Neurosci.* 9, 1234-1236. 10.1038/nn1774 16980964

[DEV204945C78] Wu, S.-C., Liao, C.-W., Pan, R.-L. and Juang, J.-L. (2012). Infection-induced intestinal oxidative stress triggers organ-to-organ immunological communication in Drosophila. *Cell Host Microbe* 11, 410-417. 10.1016/j.chom.2012.03.004 22520468

[DEV204945C79] Yang, C. and Merlin, D. (2024). Unveiling colitis: a journey through the dextran sodium sulfate-induced model. *Inflamm. Bowel Dis.* 30, 844-853. 10.1093/ibd/izad312 38280217 PMC11063560

[DEV204945C80] Yaniv, S. P. and Schuldiner, O. (2016). A fly's view of neuronal remodeling. *Wiley Interdiscip. Rev. Dev. Biol.* 5, 618-635. 10.1002/wdev.241 27351747 PMC5086085

[DEV204945C81] Yu, C., An, Z., Zhao, W., Wang, W., Gao, C., Liu, S., Wang, J. and Wu, J. (2015). Sex differences in stroke subtypes, severity, risk factors, and outcomes among elderly patients with acute ischemic stroke. *Front. Aging Neurosci.* 7, 174. 10.3389/fnagi.2015.00174 26441636 PMC4561826

